# Sustainable grassland systems: a modelling perspective based on the North Wyke Farm Platform

**DOI:** 10.1111/ejss.12304

**Published:** 2015-11-17

**Authors:** L. Wu, X. Zhang, B. A. Griffith, T. H. Misselbrook

**Affiliations:** ^1^ Sustainable Soils and Grassland Systems DepartmentRothamsted ResearchNorth WykeOkehamptonEX20 2SBUK; ^2^ Ministry of Agriculture Key Laboratory of Crop Nutrition and FertilizationInstitute of Agricultural Resources and Regional Planning, Chinese Academy of Agricultural Sciences12 Zhongguancun South Avenue, Beijing100081China

## Abstract

The North Wyke Farm Platform (NWFP) provides data from the field‐ to the farm‐scale, enabling the research community to address key issues in sustainable agriculture better and to test models that are capable of simulating soil, plant and animal processes involved in the systems. The tested models can then be used to simulate how agro‐ecosystems will respond to changes in the environment and management. In this study, we used baseline datasets generated from the NWFP to validate the Soil‐Plant‐Atmosphere Continuum System (SPACSYS) model in relation to the dynamics of soil water content, water loss from runoff and forage biomass removal. The validated model, together with future climate scenarios for the 2020s, 2050s and 2080s (from the International Panel on Climate Change (IPCC) Special Report on Emissions Scenarios (SRES): medium (A1B) and large (A1F1) emission scenarios), were used to simulate the long‐term responses of the system with three contrasting treatments on the NWFP. Simulation results demonstrated that the SPACSYS model could estimate reliably the dynamics of soil water content, water loss from runoff and drainage, and cut biomass for a permanent sward. The treatments responded in different ways under the climate change scenarios. More carbon (C) is fixed and respired by the swards treated with an increased use of legumes, whereas less C was lost through soil respiration with the planned reseeding. The deep‐rooting grass in the reseeding treatment reduced N losses through leaching, runoff and gaseous emissions, and water loss from runoff compared with the other two treatments.

## Introduction

The North Wyke Farm Platform (NWFP), a farm‐scale research platform for grassland‐based beef and sheep production, was established during 2010 in southwest England (50°46′10″N, 3°54′05″W). It is a United Kingdom National Capability funded by the Biotechnology and Biological Sciences Research Council (BBSRC) for collaborative research, training and knowledge exchange in agro‐environmental science; it addresses agricultural productivity and ecosystem responses to different management practices. One of the purposes of the NWFP is to test existing models with high spatial and temporal resolution data.

The global average surface temperature has increased by about 0.7°C in the last century and is projected to increase by another 1.1–6.4°C in this century. Long‐term trends in the amounts of precipitation were spatially variable during the last century and the amounts are likely to increase at high latitudes and decrease in most subtropical land regions during this century (IPCC, 2007). Climate change might affect the decomposition of organic matter in soil and other biogeochemical processes, such as the nitrogen (N) cycle, that might cause diffuse water pollution or greenhouse gas (GHG) emissions. Because the climate also controls the processes of plant growth and development, plant response to climate change is not determined solely by photosynthesis, but also by the partitioning of photosynthate among plant organs and the progress of its development.

The Soil‐Plant‐Atmosphere Continuum System (SPACSYS) model (Wu *et al.*, 2007) is a field‐scale, weather‐driven, process‐based and daily‐time‐step dynamic model to simulate plant growth and development, soil N and carbon (C) cycling, soil water movement and heat transformation in multiple and isolated (no energy and matter exchange between fields) fields simultaneously. The model has been used to investigate several issues, including nitrate leaching and efficient use of resources by crops and root systems. The model can also be used to assess field management options to mitigate greenhouse gas emissions and diffuse pollution and the effects that climate change might have on plant performance, C sequestration and mitigation potential.

Our hypothesis is that the projected climate change will affect soil processes such as water movement and nutrient cycling, with subsequent effects on plant productivity. In this study, we used partial datasets generated between April 2011 and May 2014 from the NWFP to validate the SPACSYS model in forage biomass removal, runoff flow and the dynamics of soil water content. Next, we used the validated model together with future climate projections to simulate the long‐term responses of the systems with the three management systems implemented on the NWFP.

## Methods and materials

### *Farm platform*

The mean annual rainfall between 1982 and 2011 at the North Wyke site was 1042 mm, with an annual average temperature that ranged from 6.6 to 13.4°C. North Wyke has relatively large (compared with the UK average) and consistent amounts of summer rainfall, which is characteristic of the major agricultural grassland areas in the west of the UK. The environmental conditions are sufficient to support 280 days of grass growth annually, but the grazing season is often restricted to 180 days because of soil wetness.

**Figure 1:**
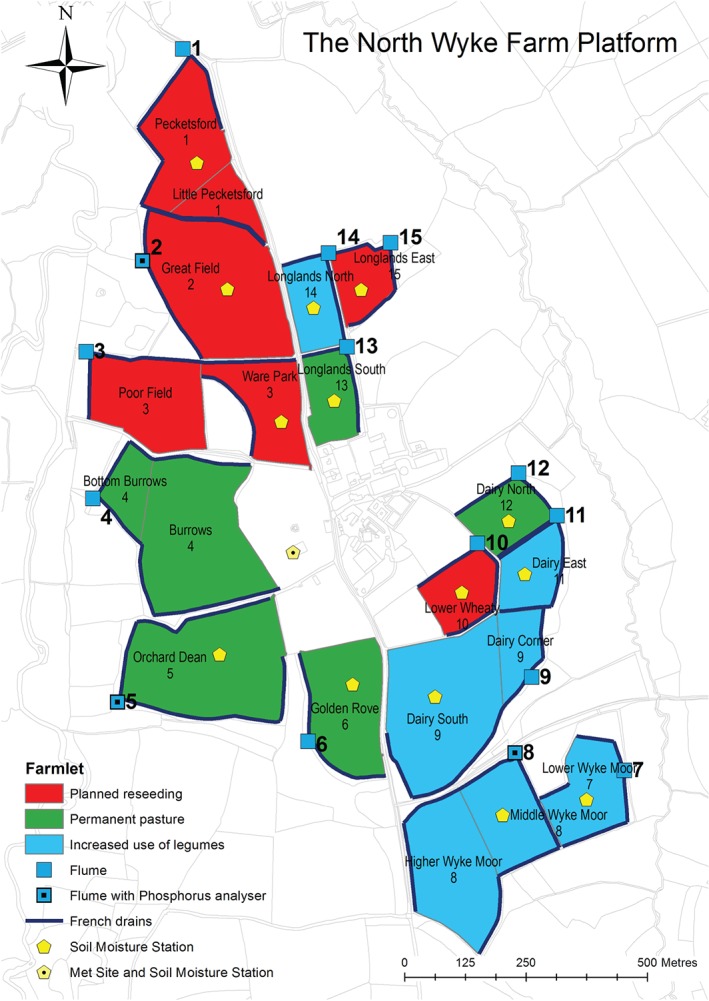
The North Wyke Farm Platform (the flume number is indicated next to each flume symbol).

The NWFP comprises three farmlets of approximately 22 ha each in size, which are designed to test the productivity and environmental sustainability of contrasting temperate grassland systems for beef and sheep at appropriate farm scales. Each of the three farmlets consists of five sub‐catchments (15 in total, Figure 1), where all water runoff from each sub‐catchment is channelled through a single flume (an outlet at which measurements can be made). At each flume, water chemistry (the concentrations of nitrate, ammonium, dissolved ammonia and dissolved organic carbon, dissolved oxygen, water turbidity and pH value), water temperature and water flow are recorded at 15‐minute intervals. More details of the system are given by Orr *et al.* (2011).

In a 2‐year period from 1 April 2011 to 31 March 2013, beef and sheep systems were managed in the same way on the three farmlets to measure baseline productivity on the existing permanent pasture. From 1 April 2013, new types of management were established progressively on two of the farmlets, giving three contrasting treatments: (i) sustainable intensification of permanent grassland (mineral N fertilizer application, maintaining current practice), (ii) increased use of legumes (introduction of legumes, mixing the cultivars AberMagic (*Lolium perenne* L.) and AberHerald (*Trifolium repens* L.) in two fields of the farmlet in 2013) and (iii) planned reseeding (planned reseeding, resowing the cultivar Prior (*Festulolium loliaceum*), a deep‐rooting grass, in a field of the farmlet in 2013). Six fields that drain to five flumes were chosen randomly from those where the treatments were being conducted. Three fields for the planned reseeding treatment were selected: one was kept under current management and the other two, which share the same flume, were in transition. Two fields were selected for the introduction of legumes: one was kept under current management and the other was in transition. The data obtained from these fields between April 2011 and May 2014 were used for this study (Table 1).

**Table 1:** Farm platform fields and flumes used

Treatment	Flume number	Field name
Sustainable intensification	6	Golden Rove
Increased use of legumes	8	Higher Wyke Moor and Middle Wyke Moor (reseeding in 2013)
	11	Dairy East
Planned reseeding	10	Lower Wheaty
	15	Longlands East (reseeding in 2013)

### *Model description*

The main processes that concern plant growth in the model are plant development, assimilation, respiration, water and N uptake, partitioning of photosynthate and absorbed N, N fixation for legume plants and root growth. Nitrogen cycling coupled with C cycling in the model covers the transformation processes for organic matter (OM) and inorganic N. The organic matter pool is divided further into fresh OM, dissolved OM, a litter pool and a humus pool, and inorganic N includes a nitrate pool and an ammonium pool. The main processes and transformations that result in changes in size of the soluble N pools are mineralization, nitrification, denitrification and plant N uptake. Nitrate is transported through the soil profile and into field drains or deep groundwater by water movement. A biologically based component for the processes of nitrification and denitrification has been implemented in the model that can estimate gaseous N emissions. The Richards equation for water potential and Fourier's equation for temperature are used to simulate water and heat fluxes.

### *Data input and parameterization*

To mimic grazing systems, daily grass intake and excretion of sheep and cattle in the field were quantified based on literature and experimental results from our site. Detailed information that is used to estimate inputs to various N pools in the model is given in Tables 2, 3, 4. As the model does not simulate volatilization, N input from excretion was reduced by a proportion equivalent to 0.6 of the total N in the farm‐yard manure (FYM) without considering the effect of temperature on the volatilization process. It is difficult to model individual animals in the field so we assumed a live weight of 600 kg per animal for beef cattle, 75 kg per animal for ewes and, for fields that were grazed, a spatially uniform distribution of grass intake over the grazing period.

**Table 2:** Parameters of daily grass intake by sheep and beef cattle

		References
Bites / bites day^−1^		
Beef cattle	25 220	Orr *et al.* (2014)
Sheep	33 480	Gibb & Orr (1997)
Mass amount / g DM per bite		
Beef cattle	0.775	Soder *et al.* (2009)
Sheep	0.087	Gibb & Orr (1997)

**Table 3:** Daily excretion of adult beef cattle and nitrogen content

**Urine**		
Events	8.60 day^−1^	Misselbrook *et al*. (2013)
Volume	2.20 l	Misselbrook *et al*. (2013)
Area covered	0.370 m^2^	Moir *et al.* (2011)
N concentration	17.00 g N l^−1^	Misselbrook *et al*. (2013)
NH_3_ loss	0.600 fraction of total N	Based on Whitehead (1995, p. 153)
NH4	11.07 g N per event	Using urea proportion based on Whitehead (1995, p. 74)
Dissolved organic N	3.89 g N per event	—
**Faeces**		
Events	10 day^−1^	Orr *et al.* (2012)
Area coverage	0.017 m^2^	Omaliko (1981)
Dry matter	235.0 g DM per event	Orr *et al.* (2012)
N concentration	0.025 g N g^−1^ DM	Orr *et al.* (2012)
N	4.15 g per event	Orr *et al.* (2012)
NH_3_ loss	0.086 fraction of total N	McGechan & Topp (2004)
NH4	0.034 g N per event	Based on Whitehead (1995, p. 77)
Dissolved organic N	0.835 g N per event	Based on Whitehead (1995, p. 77)

**Table 4:** Daily excretion by sheep and nitrogen content

**Urine**		
Events	17 day^−1^	Wheeler (1959) and Rosen *et al.* (2004)
Volume	0.150 l	Wheeler (1959) and Rosen *et al.* (2004)
Area covered	0.030 m^2^	Wheeler (1959) and Williams & Haynes (1995)
N concentration	10.00 g N l^−1^	Whitehead (1995, p. 74)
P concentration	0.029 g P l^−1^	Shand *et al.* (2002)
NH_3_ loss	0.600 fraction of total N	Based on Whitehead (1995, p. 153)
NH4	0.050 g N per event	Sakadevan *et al.* (1993)
Dissolved organic N	0.550 g N per event	—
**Faeces**		
Events	22 day^−1^	Williams & Haynes (1995)
Area covered	0.018 m^2^	Peterson *et al.* (1956)
Dry matter	30.00 g per event	Williams & Haynes (1995)
N concentration	0.025 g N g^−1^ DM	Whitehead (1995, p. 77)
N	0.750 g per event	—
NH_3_ loss	0.086 fraction of total N	McGechan & Topp (2004)
NH4	0.003 g N per event	Sakadevan *et al.* (1993)
Dissolved organic N	0.192 g N per event	Based on Whitehead (1995, p. 77)

Soil physical and chemical properties of the selected fields were based on baseline field surveys conducted in 2012. Agronomic management quantified for the simulation was interpreted from the farm records for the NWFP. The concentrations of nutrients in applied farmyard manure were estimated based on the DEFRA fertilizer manual (Department for Environment, Food and Rural Affairs, 2010). We assumed that the reported available N content of FYM in the manual is incorporated fully into the soil without further loss.

The SPACSYS model has been parameterized previously for the processes of soil water, soil heat transformation, and C and N cycling (Wu & Shepherd, 2011). Parameters related to grass species were adopted from a previous study. Those parameters were used directly in the simulations.

The data extracted from the UK Climate Projection 2009 (UKCP09) for future climate projections were applied to this study. The UKCP09 weather generator provides probabilistic projections of climate change (Jones *et al.*, 2009). Medium (SRES A1B) and large (SRES A1F1) emission scenarios based on future projections of greenhouse gas and aerosol levels according to the IPCC (IPCC, 2007) were used to generate future climate conditions. The scenarios at the time slices of the 2020s, 2050s and 2080s were considered. One hundred files of 30‐year daily weather variables for each time‐slice under each emission scenario and a baseline representing the 1961–1990 period were generated for the site. To avoid the need for hundreds of simulations with SPACSYS, the mean daily value across the hundred files of each weather element (except precipitation) for each day of the 30 years of data was calculated. Because of its skewed distribution, daily means of precipitation across the files cannot be taken. Therefore, the monthly mean precipitation and the number of rain days per month were calculated for each file, and then both of these elements were averaged across the 100 files. The daily precipitation for a given month was then distributed randomly across the month. As wind speed is not included in UKCP09, it was obtained from the 11‐member Regional Climate Model dataset (Met Office, 2003). Six UKCP09 projections were produced, three time‐slices for medium (represented as 2020med, 2050med and 2080med, respectively) and three time‐slices for large emissions (represented 2020lar, 2050lar and 2080lar, respectively), plus historic climate data over the period 1961–1990 (symbolized as baseline) for the site. Annual mean climate characteristics for the time‐slices under the emission scenarios are given in Table 5.

**Table 5:** Annual mean climate characteristics at baseline and time‐slices at the site

	Temperature / °C		Precipitation / mm
	Maximum	Minimum	
Baseline	12.8	5.8	1029
2020 medium	14.3	7.2	1051
2050 medium	15.4	8.1	1058
2080 medium	16.4	9.0	1046
2020 large	14.3	7.1	1022
2050 large	15.7	8.3	1025
2080 large	17.5	10.0	1043

To avoid further complexity in future scenarios, the current atmospheric CO_2_ concentration (695 mg CO_2_ m^−3^) was applied to all the simulations. Meanwhile, the current farm management practices for the individual fields (e.g. timing and amount of fertilizer or slurry application, grass‐cutting dates, start and end dates of grazing and number of animals) were kept the same for all simulations in the field. Therefore, any change in the fluxes of water, N and C as a result of the treatments would be the consequence of climate change scenarios.

### *Statistical analyses*

The statistical methods suggested by Smith *et al.* (1997) were used to evaluate the performance of the model by comparing simulation results and observed data, which enabled evaluation even where the observed data were not replicated (see Figure 1 of Smith *et al.*, 1997). Seven elements were included: correlation coefficient (*r*), root mean square error (RMSE), modelling efficiency (EF), the coefficient of determination (*R* ^2^), relative error (RE), mean deviation (MD) and maximum error (ME). When an RMSE value is less than the RMSE value at the 95% confidence level, it indicates that the simulated values fall within the 95% confidence interval of the measurements. An RE value greater than the RE value at the 95% confidence level indicates that the bias in the simulation is greater than the 95% confidence interval of the measurement.

One‐way anova was applied to test for significant differences in the simulated average annual evapotranspiration between the treatments within a given time‐slice under the various climate scenarios. The significant differences among the treatments were compared with the least significant differences (LSD) at 5, 1 and 0.1% levels of probability. Statistical analysis was performed with R (R Core Team, 2013).

## Results

### *Model validation*

#### *Cutting of biomass*

**Figure 2:**
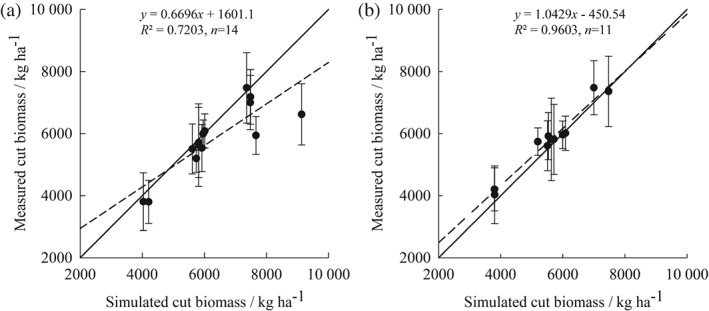
Comparison of simulated and observed cut biomass over the simulated period for the NWFP: (a) with and (b) without that cut after reseeding. Dashed line shows the fitted relationship; solid line is 1:1 line and error bars are standard deviations for observed data.

Simulated cutting of biomass from all the fields selected over the period of simulation was compared with observed data (Figure 2). The simulated cutting of biomass agrees well with the sampled data when samples taken during the establishment period in the reseeding fields were excluded. Statistical analysis also suggests that the simulations fit the measured data reasonably well (Table 6). Simulated values follow the same pattern as measured values (significant association) and describe the trend in the measured data better than the mean of the observations (positive value for EF). Furthermore, the RE value is within the 95% confidence interval of the data, indicating no bias. However, another indicator of model bias, MD, shows a significant bias towards over‐estimation.

**Table 6:** Statistical analysis of simulated and measured cut biomass

	*r*	RMSE (RMSE_95%_)	EF	*R* ^2^	RE (RE_95%_)	MD	ME	Number of samples
Including data from first year of establishment	0.85*	15 (77)	0.34	0.62	−8 (65)	−481	2 509	14
Excluding data from first year of establishment	0.98*	5 (86)	0.92	1.13	−4 (73)	−200	543	11

* Significant association at 5% level.

RMSE, root mean square error; RMSE_95%_, RMSE at the 95% confidence level; EF, modelling efficiency; RE, relative error; RE_95%_, RE at the 95% confidence level; MD, mean deviation; ME, maximum error.

#### *Soil moisture*

**Figure 3:**
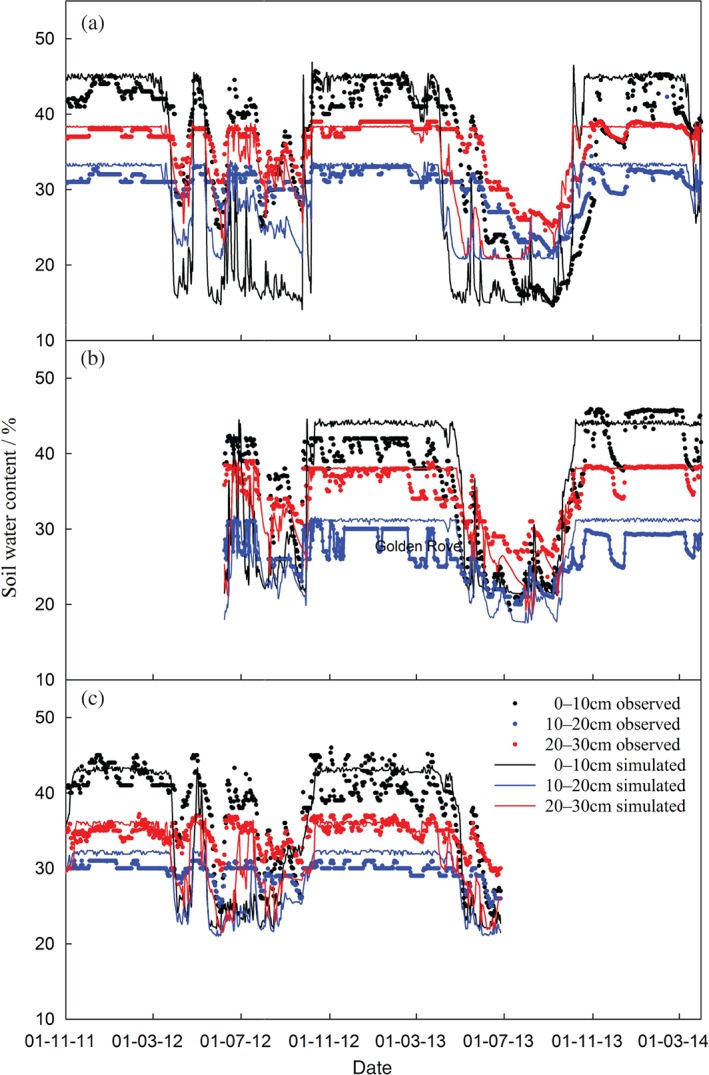
Comparison of measured (solid circle) and simulated (solid line) soil moisture at different soil depths for (a) Lower Wheaty, (b) Golden Rove and (c) Middle Wyke Moor fields.

Simulated soil water content at different soil depths follows the same trends as the observed data (Table 7), especially when the soil samples are approaching saturation. The comparisons between simulated and observed data for the Lower Wheaty, Golden Rove and Middle Wyke Moor fields for the periods when observed data were available are shown in Figure 3. Results are similar for the rest of the fields (not shown). There is a large discrepancy between simulated and observed water content in the topsoil layer, particularly during periods of drought. The vertical distribution of soil water content depends on the soil's physical properties, whereas its temporal distribution is affected by precipitation and grass growth. For example, soil water decreased dramatically in the top 30 cm of the soil profiles in the summer of 2013 because of less precipitation and vigorous growth of grass.

**Table 7:** Statistical analysis of model's performance on dynamics of soil moisture for different treatments

Criteria	Control	AN	Slurry
*r*	0.77*	0.76*	0.78*
RMSE (RMSE_95%_)	17 (67)	19 (47)	19 (50)
EF	0.58	0.51	0.48
*R* ^2^	1.82	0.91	0.92
RE (RE_95%_)	−3.05 (43)	2.78 (37)	5.87 (37)
MD	−1.11	0.99	2.17
ME	16.58	19.60	20.49
Number of samples	108		

* Significant association at 5% level.

RMSE, root mean square error; RMSE_95%_, RMSE at the 95% confidence level; EF, modelling efficiency; RE, relative error; RE_95%_, RE at the 95% confidence level; MD, mean deviation; ME, maximum error.

### *Surface runoff and drainage*

**Figure 4:**
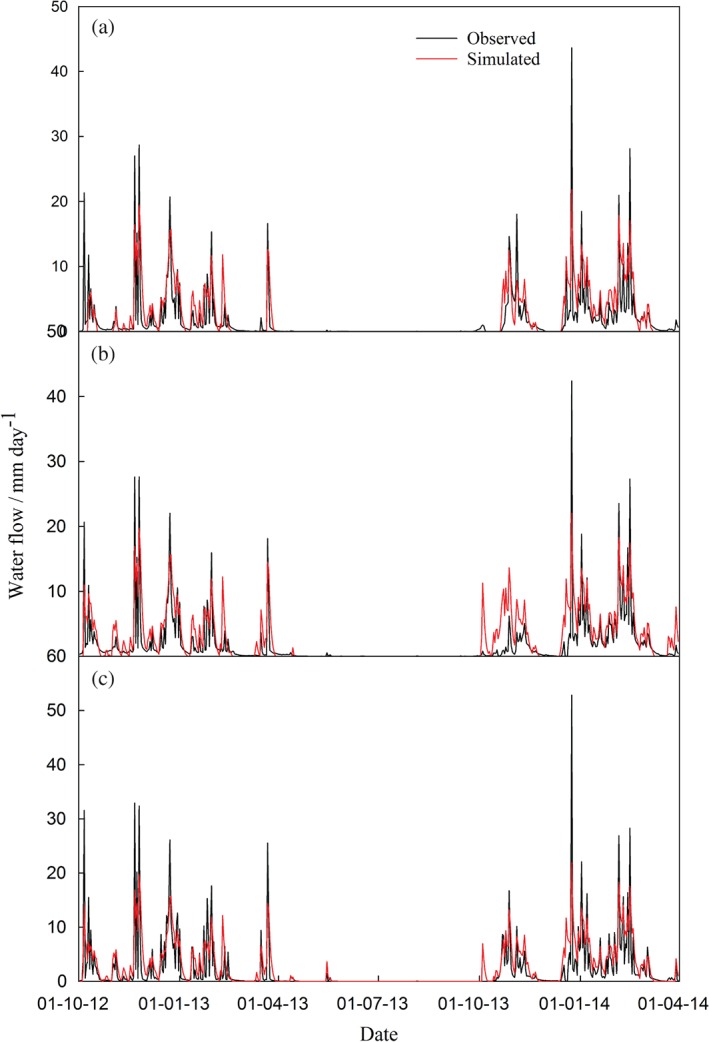
Comparison of simulated and observed water flux at (a) flume 10 (Lower Wheaty), (b) flume 6 (Golden Rove) and (c) flume 8 (Middle Wyke Moor).

A comparison between simulated water fluxes and observed data for flumes 10, 6 and 8 is shown in Figure 4 (not shown for the rest of the fields). The simulated water flux matches the pattern of observed data, with a correlation coefficient of *r* > 0.79 in all cases. All of the observed peak flow events were identified by the model. The simulations describe the trend in the measured data better than the mean of the observations (positive values for EF and *R* ^2^ close to 1) and show no significant bias towards over‐ or under‐estimation.

### *System performance with future climate projection*

**Figure 5:**
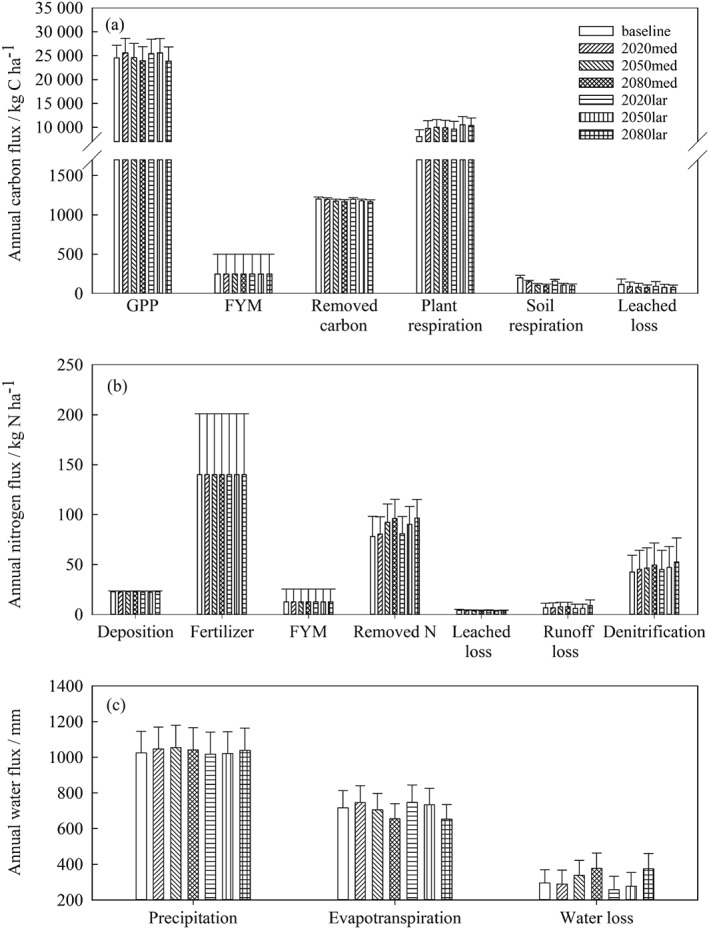
Average annual fluxes of (a) carbon, (b) nitrogen and (c) water for Golden Rove at baseline and under different climate projections. GPP, gross primary productivity; FYM, farmyard manure.

Average simulated annual fluxes of water, C and N over a 30‐year period in a field under the different climate projections vary (Tables S1–S3), as shown in Figure 5 for Golden Rove field. For all climate projections, a large proportion of grass fixed C is emitted to the atmosphere by plant respiration, with the next largest removal being that through either grazing or cut forage. About 1% only of total C removed from the system is leached. In all the climate scenarios, some of the fixed C will sequester into the soil but the capacity diminishes with time. Furthermore, the difference in the amount of sequestered C between the emission scenarios in the 2080s might disappear. However, most of the N input into the system is recovered by the grass. Among the N lost, runoff is an important component for this field. There is no significant difference in annual evapotranspiration between the emission scenarios for the same time‐slice (Table 8). The amount of evapotranspiration tends to decline with time, whereas water loss through the soil increases with time.

**Table 8:** Analysis of variance (anova) of annual evapotranspiration for the emission scenarios for the same time slice

	Source	Sum of squares	Degrees of freedom	Mean square	*F*	*P‐*value
2020 medium versus 2020 large	Between scenarios	52.9	1	52.9	0.01	0.93
	Within scenarios	409 757.7	58	7064.8		
	Total	409 810.6	59			
2050 medium versus 2050 large	Between scenarios	8047.9	1	8047.9	1.24	0.27
	Within scenarios	377 047.8	58	6500.8		
	Total	385 095.6	59			
2080 medium versus 2080 large	Between scenarios	3.0	1	3.0	0.00	0.98
	Within scenarios	318 997.9	58	5500.0		
	Total	319 000.8	59			

**Figure 6:**
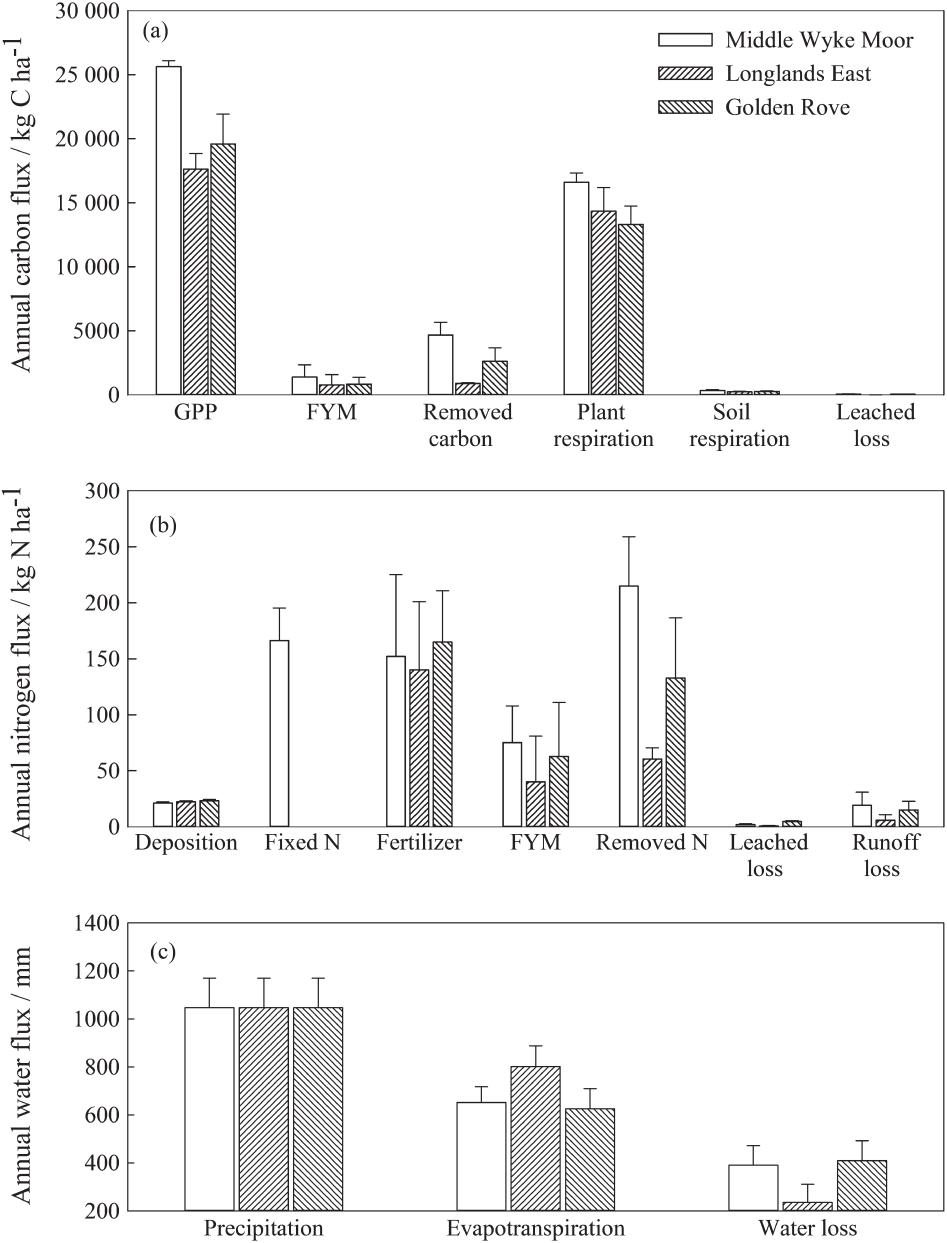
Average annual fluxes of (a) carbon, (b) nitrogen and (c) water from the three treatments under the medium scenario (SRES A1B) for the 2020s. (GPP, gross primary productivity; FYM, farmyard manure).

The responses of the three treatments to a future climate projection are different (Tables S1–S3). Figure 6 shows the average annual fluxes of water, C and N from the treatments under the medium scenario (SRES A1B) for the 2020s. More C is fixed and respired by the swards treated with the increased use of legumes. The smallest loss of C through soil respiration and leaching occurs with the planned reseeding. The increased use of legumes receives the largest N input (including deposition, fertilizer, FYM and biological N fixation) and removes the largest amount of N through sward offtake. Importantly, the deep‐rooting grass from the planned reseeding treatment reduces N losses that occur through leaching, runoff or gaseous emissions compared with those from the other two treatments. The smallest annual water loss from runoff and the largest amount of evapotranspiration from the planned reseeding field demonstrate the importance of deep rooting in water cycling.

## Discussion

The model simulates the dynamics of soil water content, water fluxes collected at the flumes and biomass removal well statistically. Although the livestock systems were simplified in the simulation, the model can still mimic the key outputs of real systems correctly. However, the current simulation of grazing, in particular, could be improved as discussed below.

Soil hydraulic properties in the different soil layers have important effects on water movement and soil water content (Baroni *et al.*, 2013) and, as a consequence, on soil heat flux (Seemann, 1979), soil C and N cycling (Milne *et al.*, 2011) and plant growth (Valentine *et al.*, 2012), and therefore the whole livestock production system. It was suggested that the spatial variation in soil hydraulic properties contributes partly to the variation in soil moisture (Hupet & Vanclooster, 2002), and that soil moisture and soil properties might have a dominant effect on catchment runoff (Merz & Plate, 1997). Currently, measurement of the physical properties of soil for the NWFP is incomplete. Measurements of these properties for the different soil layers for each field are required to test the models fully.

The simulation results show that the model estimated cut biomass reliably for a permanent sward (Figure 2b), but it overestimates it during sward establishment following reseeding. This overestimation might be a result of the partitioning coefficients of daily photosynthate. In the model the coefficients for perennial grasses are determined by a development index (Wu *et al.*, 2007) that is calculated with accumulated temperature from the sowing or cutting date and the same values are assumed for grass at the establishment stage or regrowth. In reality, photosynthate might be allocated to the roots more during the establishment period than at the regrowth stage, which could restrict canopy expansion. Previous research has shown that partitioning of C might be affected by N deficiency and regrowth (Bélanger *et al.*, 1994). The cultivar used in the planned reseeding treatment was grown at the site in a previous study. The annual average cut biomass over the period 2007–2009, after its establishment, was 9718 kg DM ha^−1^ (Macleod *et al.*, 2013), whereas our simulated cut biomass in 2014 was 9131 kg DM ha^−1^ with a relative error of 6%. Although there is some overestimation during the establishment phase, simulation gives realistic values for the post‐establishment phase; therefore, the estimation of grass biomass with this treatment can be considered reliable for future climate predictions.

Our simulation demonstrates that a mixture of grass and clover could increase C fixation and N offtake, which would improve the quality of the sward under the current management practices and potentially result in a greater livestock output. The result can be verified by field experiments conducted under different climate conditions and for different types of soil (e.g. Nyfeler *et al.*, 2011; Sturludóttir *et al.*, 2014). However, there is a risk of increased C loss through soil respiration and leaching. Kell (2011) suggested that deeper and bushy root ecosystems could improve simultaneously both soil structure and retention of its steady‐state C, water and nutrients. Our simulation of the reseeding treatment supports this because it shows smaller amounts of C leakage and N losses compared with the fertilizer treatment. Because the treatments have been established for just 1 year, by the end date of the simulations the systems had not yet stabilized after the soil disturbance caused by reseeding. More data over a longer time period are required, therefore, to verify the results of our simulations.

Our simulation results for gross primary productivity suggest less effect from the future climate projections. Given the condition that annual precipitation does not change, but that temperatures increase at the site (Table 5), warming might cause the decline of primary productivity of grasses grown in clay soil because of potential water stress. The future climate was characterized by less precipitation and higher temperatures in summer (data not shown), which would frequently trigger water stress. Therefore, a warmer climate might not increase productivity of the grasses. The simulated results are supported by a warming experiment on grass biomass production (De Boeck *et al.*, 2008). In addition, the effects of climate change, in terms of precipitation and temperature, on biomass production could have considerable seasonal and interannual variations (Bloor *et al.*, 2010). Our conclusion is based on the assumption that the cultivars (and associated traits) do not change throughout the simulation period. However, a mixture of forage species with respect to drought tolerance and increasing water‐use efficiency could improve adaptation to climate change. Hence, the interactions between climate change and the genetic traits of forage species need to be investigated further. In our study, the practices for individual fields were fixed within each time‐slice to investigate the effect of climate change on C and N cycling and water movement. Both the increased legume and planned reseeding treatments are shown to be sustainable under the future climate projections. Our simulations show that deep‐rooting grass would be beneficial for reducing surface runoff and drainage and in sequestering C into the soil, which the measurements made at the site supported. We concluded that *Festulolium* reduces water runoff from grassland and provides high‐quality forage with resilience to weather extremes. It also produced both the largest and most extensively distributed root system compared with other cultivars (Macleod *et al.*, 2013). In practice, however, farmers would adapt to climatic variability and future climate change by changing agronomic management practices. Further simulations should be carried out to assess these dynamic changes in practice (for example with regard to stocking density and start and end dates for grazing).

The current version of SPACSYS does not have a component that simulates grazing and the live‐weight gain of animals, although it can mimic the grazing process by manipulating the management practices manually (i.e. sward biomass removed daily and nutrient input from excreta). Future development of the model should focus on improving the animal component and on linking the processes involved in the soil, plant and atmosphere with livestock production, including a livestock housing component. Data available from the NWFP can be used in this context. Improved characterization of the form of excreta (urine or dung) and its spatial distribution from grazing livestock is required as these can be important factors that affect subsequent losses or transformations of N (Yamulki *et al.*, 1998). Better representation of ammonia (NH_3_) volatilization from N fertilizer and livestock manure throughout the management system is also required. Misselbrook *et al.* (2004) suggested that NH_3_ losses would usually account for about 2% of ammonium nitrate fertilizer N input and, as the available N content in FYM is typically between 10 and 25% of total N content, then the typical NH_3_ loss from FYM applied to land would be in the range 5–20% of total N applied (Nicholson *et al.*, 2013).

## Conclusion

The SPACSYS model provides good simulations on the dynamics of soil water content, water fluxes and grass biomass removal for the current treatments of the NWFP. Although the livestock systems were simplified in the simulation, it could still mimic the important outputs of real systems correctly.

The simulations demonstrated that deep‐rooting grass would be beneficial in reducing surface runoff and drainage and in sequestering C into the soil, and that the mixture of grass and legumes could increase the fixation of C and N offtake. This would improve the quality of the sward under the current management practices and potentially result in a greater livestock output. More C would be fixed and respired by the swards treated with the increased use of legumes, and the smallest C loss through soil respiration would be from the planned reseeding under the future climate projections. Both the increased legume and planned reseeding treatments would be sustainable provided that the practices for individual fields do not change with time. Our results suggest that the projected climate change would affect soil processes, with subsequent effects on plant productivity.

## Supporting information


**Table S1.** Carbon annual fluxes (kg C ha^−1^) in the simulated fields at baseline and under the various climate projections (values in parentheses are standard deviations).

**Table S2.** Nitrogen annual fluxes (kg N ha^−1^) in the simulated fields at baseline and under the various climate projections (values in parentheses are standard deviations).

**Table S3.** Water annual fluxes (mm ha^−1^) in the simulated fields at baseline and under the various climate projections (values in parentheses are standard deviations).

## References

[ejss12304-bib-0001] Baroni, G. , Ortuani, B. , Facchi, A. & Gandolfi, C. 2013 The role of vegetation and soil properties on the spatio‐temporal variability of the surface soil moisture in a maize‐cropped field. Journal of Hydrology, 489, 148–159.

[ejss12304-bib-0002] Bélanger, G. , Gastal, F. & Warembourg, F.R. 1994 Carbon balance of tall fescue (*Festuca arundinacea* Schreb.): effects of nitrogen fertilization and the growing season. Annals of Botany, 74, 653–659.

[ejss12304-bib-0003] Bloor, J.M.G. , Pichon, P. , Falcimagne, R. , Leadley, P. & Soussana, J.‐F. 2010 Effects of warming, summer drought, and CO_2_ enrichment on aboveground biomass production, flowering phenology, and community structure in an upland grassland ecosystem. Ecosystems, 13, 888–900.

[ejss12304-bib-0004] De Boeck, H.J. , Lemmens, C.M.H.M. , Zavalloni, C. , Gielen, B. , Malchair, S. , Carnol, M. *et al.* 2008 Biomass production in experimental grasslands of different species richness during three years of climate warming. Biogeosciences, 5, 585–594.

[ejss12304-bib-0005] Department for Environment Food and Rural Affairs 2010 Fertiliser Manual, Reference Book 209, 8th edn. The Stationery Office, London.

[ejss12304-bib-0006] Gibb, M. & Orr, R. 1997 Grazing behaviour of ruminants In: IGER Innovations 1 (ed Gordon A.J.), pp. 54–57. IGER, Aberystwyth.

[ejss12304-bib-0007] Hupet, F. & Vanclooster, M. 2002 Intraseasonal dynamics of soil moisture variability within a small agricultural maize cropped field. Journal of Hydrology, 261, 86–101.

[ejss12304-bib-0008] IPCC 2007 Climate Change 2007: The Physical Science Basis. Contribution of Working Group I to the Fourth Assessment Report of the Intergovernmental Panel on Climate Change. Cambridge University Press, Cambridge, U. K.

[ejss12304-bib-0009] Jones, P.D. , Kilsby, C.G. , Harpham, C. , Glenis, V. & Burton, A. 2009 UK Climate Projections Science Report: Projections of Future Daily Climate for the UK from the Weather Generator. University of Newcastle, Newcastle.

[ejss12304-bib-0010] Kell, D.B. 2011 Breeding crop plants with deep roots: their role in sustainable carbon, nutrient and water sequestration. Annals of Botany, 108, 407–418. 21813565 10.1093/aob/mcr175 PMC3158691

[ejss12304-bib-0011] Macleod, C.J.A. , Humphreys, M.W. , Whalley, W.R. , Turner, L. , Binley, A. , Watts, C.W. *et al.* 2013 A novel grass hybrid to reduce flood generation in temperate regions. Science Report, 3, doi: 10.1038/srep01683. 10.1038/srep01683 PMC3635218 23619058

[ejss12304-bib-0012] McGechan, M.B. & Topp, C.F.E. 2004 Modelling environmental impacts of deposition of excreted nitrogen by grazing dairy cows. Agriculture, Ecosystems & Environment, 103, 149–164.

[ejss12304-bib-0013] Merz, B. & Plate, E.J. 1997 An analysis of the effects of spatial variability of soil and soil moisture on runoff. Water Resources Research, 33, 2909–2922.

[ejss12304-bib-0014] Met Office 2003 Climate Impacts LINK Project.[WWW document]. URL http://badc.nerc.ac.uk/view/badc.nerc.ac.uk__ATOM__dataent_linkdata [accessed on 18 May 2015].

[ejss12304-bib-0015] Milne, A.E. , Haskard, K.A. , Webster, C.P. , Truan, I.A. , Goulding, K.W.T. & Lark, R.M. 2011 Wavelet analysis of the correlations between soil properties and potential nitrous oxide emission at farm and landscape scales. European Journal of Soil Science, 62, 467–478.

[ejss12304-bib-0016] Misselbrook, T.H. , Sutton, M.A. & Scholefield, D. 2004 A simple process‐based model for estimating ammonia emissions from agricultural land after fertilizer applications. Soil Use & Management, 20, 365–372.

[ejss12304-bib-0040] Misselbrook, T. , Umstatter, C. , Duthie, C.‐A ., Nicoll, L. , & Waterhouse, T . 2013 Automated monitoring of urination events from grazing beef cows . In: Precision Livestock Farming '13. (eds. Berckmans D. & Vandermeulen J.), Leuven, Belgium.

[ejss12304-bib-0017] Moir, J.L. , Cameron, K.C. , Di, H.J. & Fertsak, U. 2011 The spatial coverage of dairy cattle urine patches in an intensively grazed pasture system. The Journal of Agricultural Science, 149, 473–485.

[ejss12304-bib-0018] Nicholson, F.A. , Bhogal, A. , Chadwick, D. , Gill, E. , Gooday, R.D. , Lord, E. *et al.* 2013 An enhanced software tool to support better use of manure nutrients: MANNER‐NPK. Soil Use & Management, 29, 473–484.

[ejss12304-bib-0019] Nyfeler, D. , Huguenin‐Elie, O. , Suter, M. , Frossard, E. & Lüscher, A. 2011 Grass‐legume mixtures can yield more nitrogen than legume pure stands due to mutual stimulation of nitrogen uptake from symbiotic and non‐symbiotic sources. Agriculture, Ecosystems & Environment, 140, 155–163.

[ejss12304-bib-0020] Omaliko, C.P.E. 1981 Dung deposition, breakdown and grazing behavior of beef cattle at two seasons in a tropical grassland ecosystem. Journal of Range Management, 34, 360–362.

[ejss12304-bib-0021] Orr, R.J. , Griffith, B.A. , Rose, S. , Hatch, D.J. , Hawkins, J.M.B. & Murray, P.J. 2011 Designing and creating the North Wyke Farm Platform In: Catchment Science 2011 (eds Haygarth P. & Jordan P.), p. 35 Teagasc/Defra, Wexford.

[ejss12304-bib-0022] Orr, R.J. , Griffith, B.A. , Cook, J.E. & Champion, R.A. 2012 Ingestion and excretion of nitrogen and phosphorus by beef cattle under contrasting grazing intensities. Grass & Forage Science, 67, 111–118.

[ejss12304-bib-0023] Orr, R.J. , Tallowin, J.R.B. , Griffith, B.A. & Rutter, S.M. 2014 Effects of livestock breed and rearing experience on foraging behaviour of yearling beef cattle grazing unimproved grasslands. Grass & Forage Science, 69, 90–103.

[ejss12304-bib-0024] Peterson, R.G. , Lucas, H.L. & Woodhouse, W.W.J. 1956 The distribution of excreta by freely grazing cattle and its effect on pasture fertility. I. Excretal distribution. Agronomy Journal, 48, 440–444.

[ejss12304-bib-0025] R Core Team 2013 R: A Language and Environment for Statistical Computing. R Foundation for Statistical Computing, Vienna.

[ejss12304-bib-0026] Rosen, M.R. , Reeves, R.R. , Green, S. , Clothier, B. & Ironside, N. 2004 Prediction of groundwater nitrate contamination after closure of an unlined sheep feedlot. Vadose Zone Journal, 3, 990–1006.

[ejss12304-bib-0027] Sakadevan, K. , Mackay, A.D. & Hedley, M.J. 1993 Influence of sheep excreta on pasture uptake and leaching losses of sulphur, nitrogen and potassium from grazed pastures. Australian Journal of Soil Research, 31, 151–162.

[ejss12304-bib-0028] Seemann, J. 1979 Heat flux in the soil In: Agrometeorology (eds Seemann J., Chirkov Y.I., Lomas J. & Primault B.), pp. 35–37. Springer‐Verlag, Berlin.

[ejss12304-bib-0029] Shand, C.A. , Williams, B.L. , Dawson, L.A. , Smith, S. & Young, M.E. 2002 Sheep urine affects soil solution nutrient composition and roots: differences between field and sward box soils and the effects of synthetic and natural sheep urine. Soil Biology & Biochemistry, 34, 163–171.

[ejss12304-bib-0030] Smith, P. , Smith, J.U. , Powlson, D.S. , McGill, W.B. , Arah, J.R.M. , Chertov, O.G. *et al.* 1997 A comparison of the performance of nine soil organic matter models using datasets from seven long‐term experiments. Geoderma, 81, 153–225.

[ejss12304-bib-0031] Soder, K.J. , Sanderson, M.A. , Gregorini, P. , Orr, R.J. , Rubano, M.D. & Rook, A.J. 2009 Relationship of bite mass of cattle to sward structure of four temperate grasses in short‐term grazing sessions. Grass & Forage Science, 64, 421–431.

[ejss12304-bib-0032] Sturludóttir, E. , Brophy, C. , Bélanger, G. , Gustavsson, A.M. , Jørgensen, M. , Lunnan, T. *et al.* 2014 Benefits of mixing grasses and legumes for herbage yield and nutritive value in Northern Europe and Canada. Grass & Forage Science, 69, 229–240.

[ejss12304-bib-0033] Valentine, T.A. , Hallett, P.D. , Binnie, K. , Young, M.W. , Squire, G.R. , Hawes, C. *et al.* 2012 Soil strength and macropore volume limit root elongation rates in many UK agricultural soils. Annals of Botany, 110, 259–270. 22684682 10.1093/aob/mcs118 PMC3394656

[ejss12304-bib-0034] Wheeler, J.L. 1959 The effect of sheep urine on the germination and early establishment of a common weed grass. Grass & Forage Science, 14, 55–57.

[ejss12304-bib-0035] Whitehead, D.C. 1995 Grassland Nitrogen. CAB International, Wallingford, CT and Oxford.

[ejss12304-bib-0036] Williams, P.H. & Haynes, R.J. 1995 Effect of sheep, deer and cattle dung on herbage production and soil nutrient content. Grass & Forage Science, 50, 263–271.

[ejss12304-bib-0037] Wu, L. & Shepherd, A. 2011 Special features of the SPACSYS modeling package and procedures for parameterization and validation In: Methods of Introducing System Models into Agricultural Research (eds Ahuja L.R. & Ma L.), pp. 117–154. ASA, CSSA & SSSA, Madison, WI.

[ejss12304-bib-0038] Wu, L. , McGechan, M.B. , McRoberts, N. , Baddeley, J.A. & Watson, C.A. 2007 SPACSYS: integration of a 3D root architecture component to carbon, nitrogen and water cycling – model description. Ecological Modelling, 200, 343–359.

[ejss12304-bib-0039] Yamulki, S. , Jarvis, S.C. & Owen, P. 1998 Nitrous oxide emissions from excreta applied in a simulated grazing pattern. Soil Biology & Biochemistry, 30, 491–500.

