# Supplementary material for: Sustainable grassland systems: a modelling perspective based on the North Wyke Farm Platform
Source: Eur J Soil Sci. 2015 Nov 17;67(4):397–408. doi: 10.1111/ejss.12304 (PMC5108350; doi:10.1111/ejss.12304)
Supplement: Supplementary file 1 — Table S1. Carbon annual fluxes (kg C ha−1) in the simulated fields at baseline and under the various climate projections (values in parentheses are standard deviations). [file EJSS-67-397-s001.docx]

Table S1. Carbon annual fluxes (kg C ha^-1^) in the simulated fields under the baseline and various climate projections (values in parentheses are standard deviations)

|  |  |  | Input | |  | |  | Output | | | |  |
| --- | --- | --- | --- | --- | --- | --- | --- | --- | --- | --- | --- | --- |
| Field | Climate scenario^*^ | Gross primary productivity | | Farmyard manure | | Removed biomass | | | Grass respiration | Soil respiration | Leached loss | |
| Golden Rove | Baseline | 24 522 (2650) | | 836 (542) | | 2444 (877) | | | 11 599 (1159) | 341 (42) | 54 (12) | |
|  | 2020med | 25 582 (3042) | | 836 (542) | | 2616 (1060) | | | 13 309 (1427) | 261 (44) | 46 (12) | |
|  | 2050med | 24 611 (2975) | | 836 (542) | | 2520 (971) | | | 13 386 (1335) | 221 (44) | 41 (9) | |
|  | 2080med | 23 950 (2954) | | 836 (542) | | 2371 (815) | | | 13 335 (1285) | 215 (44) | 37 (10) | |
|  | 2020lar | 25 433 (3007) | | 836 (542) | | 2567 (1002) | | | 13 150 (1374) | 271 (43) | 46 (11) | |
|  | 2050lar | 25 568 (3037) | | 836 (542) | | 2534 (981) | | | 14 067 (1403) | 224 (44) | 40 (11) | |
|  | 2080lar | 23 854 (2999) | | 836 (542) | | 2268 (712) | | | 13 630 (1341) | 215 (48) | 35 (9) | |
| Higher Wyke Moor | Baseline | 28 756 (1175) | | 765 (65) | | 2807 (1792) | | | 13 194 (973) | 331 (55) | 23 (18) | |
|  | 2020med | 29 603 (1310) | | 765 (65) | | 2890 (1884) | | | 14 848 (1323) | 255 (37) | 19 (12) | |
|  | 2050med | 29 123 (1172) | | 765 (65) | | 2841 (1840) | | | 15 358 (1216) | 213 (34) | 17 (11) | |
|  | 2080med | 29 075 (1452) | | 765 (65) | | 2740 (1741) | | | 15 790 (1277) | 208 (32) | 16 (12) | |
|  | 2020lar | 29 376 (1302) | | 765 (65) | | 2850 (1854) | | | 14 675 (1237) | 260 (41) | 19 (15) | |
|  | 2050lar | 28 918 (1464) | | 765 (65) | | 2484 (1844) | | | 16 223 (1274) | 213 (35) | 13 (13) | |
|  | 2080lar | 28 693 (1360) | | 765 (65) | | 2229 (1573) | | | 16 855 (1353) | 209 (32) | 13 (13) | |
| Middle Wyke Moor | Baseline | 32 365 (924) | | 1392 (952) | | 4439 (824) | | | 14 874 (661) | 378 (95) | 67 (18) | |
|  | 2020med | 33 467 (603) | | 1392 (952) | | 4667 (1000) | | | 16 610 (717) | 327 (80) | 52 (21) | |
|  | 2050med | 34 366 (654) | | 1392 (952) | | 4687 (1120) | | | 17 584 (638) | 302 (70) | 51 (21) | |
|  | 2080med | 34 860 (624) | | 1392 (952) | | 4556 (1146) | | | 18 433 (616) | 299 (69) | 46 (17) | |
|  | 2020lar | 33 282 (629) | | 1392 (952) | | 4649 (1013) | | | 16 451 (727) | 331 (79) | 48 (20) | |
|  | 2050lar | 34 578 (615) | | 1392 (952) | | 4650 (1121) | | | 17 886 (612) | 302 (71) | 45 (17) | |
|  | 2080lar | 35 132 (569) | | 1392 (952) | | 4384 (1121) | | | 19 293 (557) | 302 (67) | 47 (18) | |
| Dairy East | Baseline | 15 452 (3311) | | 248 (252) | | 1201 (25) | | | 8065 (1426) | 200 (32) | 114 (69) | |
|  | 2020med | 16 641 (3379) | | 248 (252) | | 1197 (21) | | | 9775 (1628) | 149 (20) | 89 (56) | |
|  | 2050med | 16 224 (3373) | | 248 (252) | | 1174 (26) | | | 10 009 (1601) | 106 (19) | 81 (47) | |
|  | 2080med | 15 972 (3271) | | 248 (252) | | 1166 (27) | | | 9916 (1563) | 101 (17) | 76 (33) | |
|  | 2020lar | 16 641 (3348) | | 248 (252) | | 1198 (22) | | | 9657 (1572) | 158 (21) | 91 (60) | |
|  | 2050lar | 16 918 (3556) | | 248 (252) | | 1177 (23) | | | 10 540 (1725) | 108 (20) | 76 (41) | |
|  | 2080lar | 16 487 (3110) | | 248 (252) | | 1165 (26) | | | 10 392 (1576) | 100 (18) | 78 (29) | |
| Lower Wheaty | Baseline | 15 116 (2765) | | 650 (661) | | 1641 (48) | | | 7318 (1235) | 239 (35) | 61 (11) | |
|  | 2020med | 16 360 (2979) | | 650 (661) | | 1632 (48) | | | 8917 (1515) | 197 (29) | 55 (11) | |
|  | 2050med | 16 138 (2757) | | 650 (661) | | 1616 (50) | | | 9205 (1406) | 157 (30) | 54 (15) | |
|  | 2080med | 16 078 (2728) | | 650 (661) | | 1615 (49) | | | 9221 (1399) | 152 (28) | 58 (18) | |
|  | 2020lar | 16 376 (2963) | | 650 (661) | | 1634 (48) | | | 8828 (1471) | 205 (30) | 55 (10) | |
|  | 2050lar | 17 022 (3034) | | 650 (661) | | 1621 (49) | | | 9774 (1574) | 160 (32) | 56 (12) | |
|  | 2080lar | 16 525 (2640) | | 650 (661) | | 1616 (50) | | | 9671 (1439) | 152 (30) | 61 (22) | |
| Longlands East | Baseline | 21 905 (1616) | | 786 (799) | | 895 (55) | | | 12 146 (1443) | 316 (84) | 14 (6) | |
|  | 2020med | 23 005 (1612) | | 786 (799) | | 890 (57) | | | 14 343 (1847) | 229 (51) | 12 (7) | |
|  | 2050med | 23 714 (1469) | | 786 (799) | | 872 (58) | | | 15 319 (1492) | 172 (29) | 13 (11) | |
|  | 2080med | 23 838 (1342) | | 786 (799) | | 865 (59) | | | 15 428 (1352) | 166 (28) | 12 (11) | |
|  | 2020lar | 23 021 (1422) | | 786 (799) | | 891 (57) | | | 14 185 (1651) | 240 (53) | 12 (8) | |
|  | 2050lar | 23 977 (1460) | | 786 (799) | | 875 (57) | | | 15 540 (1506) | 172 (33) | 11 (10) | |
|  | 2080lar | 24 614 (1491) | | 786 (799) | | 862 (60) | | | 15 909 (1294) | 167 (26) | 14 (13) | |

^*^ baseline: historic climate; 2020med, 2050med, 2080med: projected climate for medium (SRES A1B) emission scenario based on future projections of greenhouse gas and aerosol levels according to IPCC determined storylines at 2020s, 2050s and 2080s, respectively; and 2020lar, 2050lar, 2080lar: projected climate for high (SRES A1F1) emission scenario at 2020s, 2050s and 2080s, respectively.
