# Supplementary material for: Sustainable grassland systems: a modelling perspective based on the North Wyke Farm Platform
Source: Eur J Soil Sci. 2015 Nov 17;67(4):397–408. doi: 10.1111/ejss.12304 (PMC5108350; doi:10.1111/ejss.12304)
Supplement: Supplementary file 2 — Table S2. Nitrogen annual fluxes (kg N ha−1) in the simulated fields at baseline and under the various climate projections (values in parentheses are standard deviations). [file EJSS-67-397-s002.docx]

Table S2. Nitrogen annual fluxes (kg N ha^-1^) in the simulated fields under the baseline and various climate projections (values in parentheses are standard deviations)

|  |  | Input | | | |  | Output | | | |
| --- | --- | --- | --- | --- | --- | --- | --- | --- | --- | --- |
|  |  | Deposition | Fixed N | Fertiliser | FYM |  | Grass removed | Leached loss | Surface loss | Denitrification |
| Golden Rove | Baseline | 23 (1) |  | 165 | 63 (48) |  | 116 (42) | 5 (1) | 16 (9) | 43 (17) |
|  | 2020med | 23 (1) |  | 165 | 63 (48) |  | 133 (54) | 5 (1) | 15 (8) | 45 (19) |
|  | 2050med | 23 (1) |  | 165 | 63 (48) |  | 144 (56) | 4 (1) | 14 (7) | 47 (20) |
|  | 2080med | 23 (1) |  | 165 | 63 (48) |  | 141 (49) | 4 (1) | 14 (7) | 50 (22) |
|  | 2020lar | 23 (1) |  | 165 | 63 (48) |  | 130 (51) | 5 (1) | 14 (7) | 45 (19) |
|  | 2050lar | 23 (1) |  | 165 | 63 (48) |  | 144 (56) | 4 (1) | 13 (6) | 47 (21) |
|  | 2080lar | 23 (1) |  | 165 | 63 (48) |  | 136 (42) | 4 (1) | 16 (8) | 53 (24) |
| Higher Wyke Moor | Baseline | 23 (1) | 71 (34) | 147 | 68 (11) |  | 157 (66) | 1 (3) | 14 (11) | 35 (11) |
|  | 2020med | 23 (1) | 52 (20) | 147 | 68 (11) |  | 162 (74) | 1 (2) | 15 (13) | 37 (13) |
|  | 2050med | 23 (1) | 38 (15) | 147 | 68 (11) |  | 168 (72) | 1 (2) | 15 (13) | 40 (14) |
|  | 2080med | 23 (1) | 40 (13) | 147 | 68 (11) |  | 169 (72) | 1 (2) | 13 (11) | 43 (16) |
|  | 2020lar | 22 (1) | 57 (22) | 147 | 68 (11) |  | 168 (73) | 1 (2) | 13 (11) | 37 (12) |
|  | 2050lar | 22 (1) | 8 (12) | 147 | 68 (11) |  | 159 (86) | 1 (2) | 11 (10) | 41 (14) |
|  | 2080lar | 22 (1) | 15 (10) | 147 | 68 (11) |  | 149 (79) | 1 (2) | 13 (11) | 46 (17) |
| Middle Wyke Moor | Baseline | 21 (1) | 151 (26) | 152 | 75 (33) |  | 195 (39) | 2 (1) | 17 (11) | 15 (4) |
|  | 2020med | 21 (1) | 166 (29) | 152 | 75 (33) |  | 215 (44) | 2 (1) | 19 (12) | 18 (4) |
|  | 2050med | 21 (1) | 167 (23) | 152 | 75 (33) |  | 217 (45) | 2 (1) | 19 (12) | 20 (6) |
|  | 2080med | 21 (1) | 175 (25) | 152 | 75 (33) |  | 215 (43) | 2 (1) | 18 (12) | 23 (7) |
|  | 2020lar | 21 (1) | 166 (31) | 152 | 75 (33) |  | 213 (45) | 2 (1) | 18 (12) | 18 (4) |
|  | 2050lar | 21 (1) | 173 (25) | 152 | 75 (33) |  | 218 (45) | 2 (1) | 17 (12) | 20 (6) |
|  | 2080lar | 21 (1) | 201 (27) | 152 | 75 (33) |  | 215 (43) | 2 (1) | 21 (12) | 25 (8) |
| Dairy East | Baseline | 23 (1) |  | 140 | 13 (13) |  | 78 (20) | 4 (1) | 7 (5) | 4 (1) |
|  | 2020med | 23 (1) |  | 140 | 13 (13) |  | 80 (17) | 4 (1) | 7 (5) | 4 (1) |
|  | 2050med | 23 (1) |  | 140 | 13 (13) |  | 92 (18) | 4 (1) | 8 (5) | 4 (1) |
|  | 2080med | 23 (1) |  | 140 | 13 (13) |  | 96 (19) | 4 (1) | 8 (4) | 4 (1) |
|  | 2020lar | 22 (1) |  | 140 | 13 (13) |  | 81 (17) | 4 (1) | 6 (4) | 4 (1) |
|  | 2050lar | 22 (1) |  | 140 | 13 (13) |  | 90 (18) | 3 (1) | 6 (4) | 5 (1) |
|  | 2080lar | 23 (1) |  | 140 | 13 (13) |  | 97 (18) | 4 (1) | 9 (6) | 5 (1) |
| Lower Wheaty | Baseline | 23 (1) |  | 140 | 33 (34) |  | 89 (20) | 5 (1) | 8 (5) | 45 (11) |
|  | 2020med | 23 (1) |  | 140 | 33 (34) |  | 94 (19) | 5 (1) | 7 (5) | 48 (13) |
|  | 2050med | 23 (1) |  | 140 | 33 (34) |  | 102 (17) | 5 (1) | 8 (5) | 48 (13) |
|  | 2080med | 23 (1) |  | 140 | 33 (34) |  | 103 (17) | 5 (1) | 9 (5) | 50 (13) |
|  | 2020lar | 22 (1) |  | 140 | 33 (34) |  | 93 (19) | 5 (1) | 7 (5) | 48 (13) |
|  | 2050lar | 22 (1) |  | 140 | 33 (34) |  | 100 (18) | 4 (1) | 7 (4) | 51 (14) |
|  | 2080lar | 23 (1) |  | 140 | 33 (34) |  | 102 (17) | 5 (1) | 10 (6) | 54 (14) |
| Longlands East | Baseline | 22 (1) |  | 140 | 40 (41) |  | 58 (9) | 1 (1) | 6 (6) | 3 (1) |
|  | 2020med | 22 (1) |  | 140 | 40 (41) |  | 60 (10) | 1 (1) | 6 (5) | 4 (1) |
|  | 2050med | 22 (1) |  | 140 | 40 (41) |  | 70 (13) | 1 (1) | 6 (5) | 4 (1) |
|  | 2080med | 22 (1) |  | 140 | 40 (41) |  | 73 (15) | 1 (1) | 7 (5) | 4 (1) |
|  | 2020lar | 22 (1) |  | 140 | 40 (41) |  | 60 (10) | 1 (1) | 5 (5) | 4 (1) |
|  | 2050lar | 22 (1) |  | 140 | 40 (41) |  | 68 (12) | 1 (1) | 5 (4) | 4 (1) |
|  | 2080lar | 22 (1) |  | 140 | 40 (41) |  | 75 (15) | 1 (1) | 8 (6) | 4 (1) |

^*^ baseline: historic climate; 2020med, 2050med, 2080med: projected climate for medium (SRES A1B) emission scenario based on future projections of greenhouse gas and aerosol levels according to IPCC determined storylines at 2020s, 2050s and 2080s, respectively; and 2020lar, 2050lar, 2080lar: projected climate for high (SRES A1F1) emission scenario at 2020s, 2050s and 2080s, respectively.
