# Supplementary material for: Sustainable grassland systems: a modelling perspective based on the North Wyke Farm Platform
Source: Eur J Soil Sci. 2015 Nov 17;67(4):397–408. doi: 10.1111/ejss.12304 (PMC5108350; doi:10.1111/ejss.12304)
Supplement: Supplementary file 3 — Table S3. Water annual fluxes (mm ha−1) in the simulated fields at baseline and under the various climate projections (values in parentheses are standard deviations). [file EJSS-67-397-s003.docx]

Table S3. Water annual fluxes (mm ha^-1^) in the simulated fields under the baseline and various climate projections (values in parentheses are standard deviations)

| Field | Scenario | Precipitation | Evapo-transpiration | Water runoff | Field | Scenario | Precipitation | Evapo-transpiration | Water runoff |
| --- | --- | --- | --- | --- | --- | --- | --- | --- | --- |
| Golden Rove | Baseline | 1025 (120) | 598 (82) | 414 (80) | Dairy East | Baseline | 1025 (120) | 716 (97) | 296 (75) |
|  | 2020med | 1047 (123) | 625 (84) | 409 (83) |  | 2020med | 1047 (123) | 746 (96) | 289 (79) |
|  | 2050med | 1054 (126) | 607 (80) | 434 (89) |  | 2050med | 1054 (126) | 705 (92) | 338 (84) |
|  | 2080med | 1042 (125) | 575 (74) | 455 (91) |  | 2080med | 1042 (125) | 655 (85) | 377 (86) |
|  | 2020lar | 1018 (124) | 623 (84) | 382 (84) |  | 2020lar | 1018 (124) | 747 (98) | 258 (75) |
|  | 2050lar | 1022 (122) | 630 (81) | 377 (85) |  | 2050lar | 1022 (122) | 733 (93) | 276 (78) |
|  | 2080lar | 1039 (124) | 574 (74) | 453 (89) |  | 2080lar | 1039 (124) | 653 (81) | 375 (85) |
| Higher Wyke Moor | Baseline | 1025 (120) | 672 (68) | 338 (80) | Lower Wheaty | Baseline | 1025 (120) | 683 (94) | 329 (75) |
|  | 2020med | 1047 (123) | 708 (65) | 325 (82) |  | 2020med | 1047 (123) | 713 (92) | 322 (78) |
|  | 2050med | 1054 (126) | 693 (63) | 348 (89) |  | 2050med | 1054 (126) | 671 (90) | 372 (83) |
|  | 2080med | 1042 (125) | 671 (59) | 359 (89) |  | 2080med | 1042 (125) | 624 (82) | 408 (83) |
|  | 2020lar | 1018 (124) | 707 (64) | 296 (82) |  | 2020lar | 1018 (124) | 715 (95) | 291 (76) |
|  | 2050lar | 1022 (122) | 746 (70) | 263 (81) |  | 2050lar | 1022 (122) | 698 (89) | 311 (76) |
|  | 2080lar | 1039 (124) | 706 (69) | 323 (88) |  | 2080lar | 1039 (124) | 624 (81) | 405 (85) |
| Middle Wyke Moor | Baseline | 1025 (120) | 621 (67) | 399 (78) | Longlands East | Baseline | 1025 (120) | 779 (94) | 235 (79) |
|  | 2020med | 1047 (123) | 651 (66) | 390 (82) |  | 2020med | 1047 (123) | 802 (86) | 236 (75) |
|  | 2050med | 1054 (126) | 634 (65) | 414 (91) |  | 2050med | 1054 (126) | 772 (79) | 274 (85) |
|  | 2080med | 1042 (125) | 612 (59) | 424 (91) |  | 2080med | 1042 (125) | 722 (71) | 311 (88) |
|  | 2020lar | 1018 (124) | 652 (66) | 361 (84) |  | 2020lar | 1018 (124) | 801 (89) | 206 (74) |
|  | 2050lar | 1022 (122) | 654 (66) | 362 (83) |  | 2050lar | 1022 (122) | 790 (80) | 222 (77) |
|  | 2080lar | 1039 (124) | 618 (64) | 416 (94) |  | 2080lar | 1039 (124) | 712 (72) | 319 (88) |

^*^ baseline: historic climate; 2020med, 2050med, 2080med: projected climate for medium (SRES A1B) emission scenario based on future projections of greenhouse gas and aerosol levels according to IPCC determined storylines at 2020s, 2050s and 2080s, respectively; and 2020lar, 2050lar, 2080lar: projected climate for high (SRES A1F1) emission scenario at 2020s, 2050s and 2080s, respectively.
